# Case report: Epigastric heteropagus twins and literature review

**DOI:** 10.3389/fped.2023.1088480

**Published:** 2023-04-12

**Authors:** Wenbin Zhu, Xiongjian Cui, Zhaohan Wu, Zhaolin Li, Gang Chen, Suixin Liang, Jianxiong Mao

**Affiliations:** ^1^ Department of Urology, Shenzhen Children's Hospital, China Medical University, Shenzhen, China; ^2^ Department of Neonatal Surgery, Shenzhen Children's Hospital, Shenzhen, China; ^3^ Department of Neonatal, Shenzhen Children's Hospital, Shenzhen, China

**Keywords:** heteropagus, parasitic twin, prenatal screening, omphalocele, parasites, birth defect

## Abstract

Epigastric heteropagus twins are an extremely rare congenital anomaly of conjoined twins. We present a case of epigastric heteropagus twins who were diagnosed *via* prenatal ultrasound imaging: the fetus (or host) was connected to the abdominal wall of the parasite (the dependent portion), and an omphalocele was present. The male infant was delivered by cesarean section at 35 + 5 weeks gestation. The parasite lacked a head and heart and presented long bones of the limbs. After abdominal computed tomography, omphalocele repair, and parasite removal were surgically performed under general anesthesia. After discharge (follow-up, 3 months), the infant is currently growing well and is healing satisfactorily. Forty-one cases of epigastric heteropagus twins were retrieved from database searches: 38 good postoperative outcomes, 2 perioperative deaths, and 1 termination. The case highlights that even when parasites are massive in size, births can present good outcomes with suitable surgical treatment.

## Introduction

Heteropagus twins (HT) are a rare type of conjoined twins with asymmetric sizes and developmental dysplasia with a prevalence of approximately 1 in 1 million (1). Most cases of HT are epigastric heteropagus conjoined twins (EHCT), in which the parasite (dependent portion) is joined to the upper abdomen of the autosite (host) or fetus. EHCT is characterized by the presence of a giant parasite relative to the fetus. Herein, we report a case of EHCT admitted to our hospital. We also conducted a literature review to describe the treatment of epigastric heteropagus cases with the aim of improving the understanding of this disorder and to provide summary data for related cases.

## Case report

The patient was a male infant whose mother underwent prenatal ultrasound imaging that revealed developmental abnormalities and subsequently accepted treatment at our hospital. Ultrasound examination indicated that the fetus was connected to the abdominal wall of the parasite through a channel approximately 2 cm in diameter and that an omphalocele was present. The parasite lacked a head and heart and presented the long bones of the limbs. Fetal karyotyping, chromosomal microarray analysis, and whole exome sequencing after amniocentesis did not indicate any clear abnormalities in the fetus. The infant was delivered at 35 weeks gestation by cesarean section.

**Figure 1:**
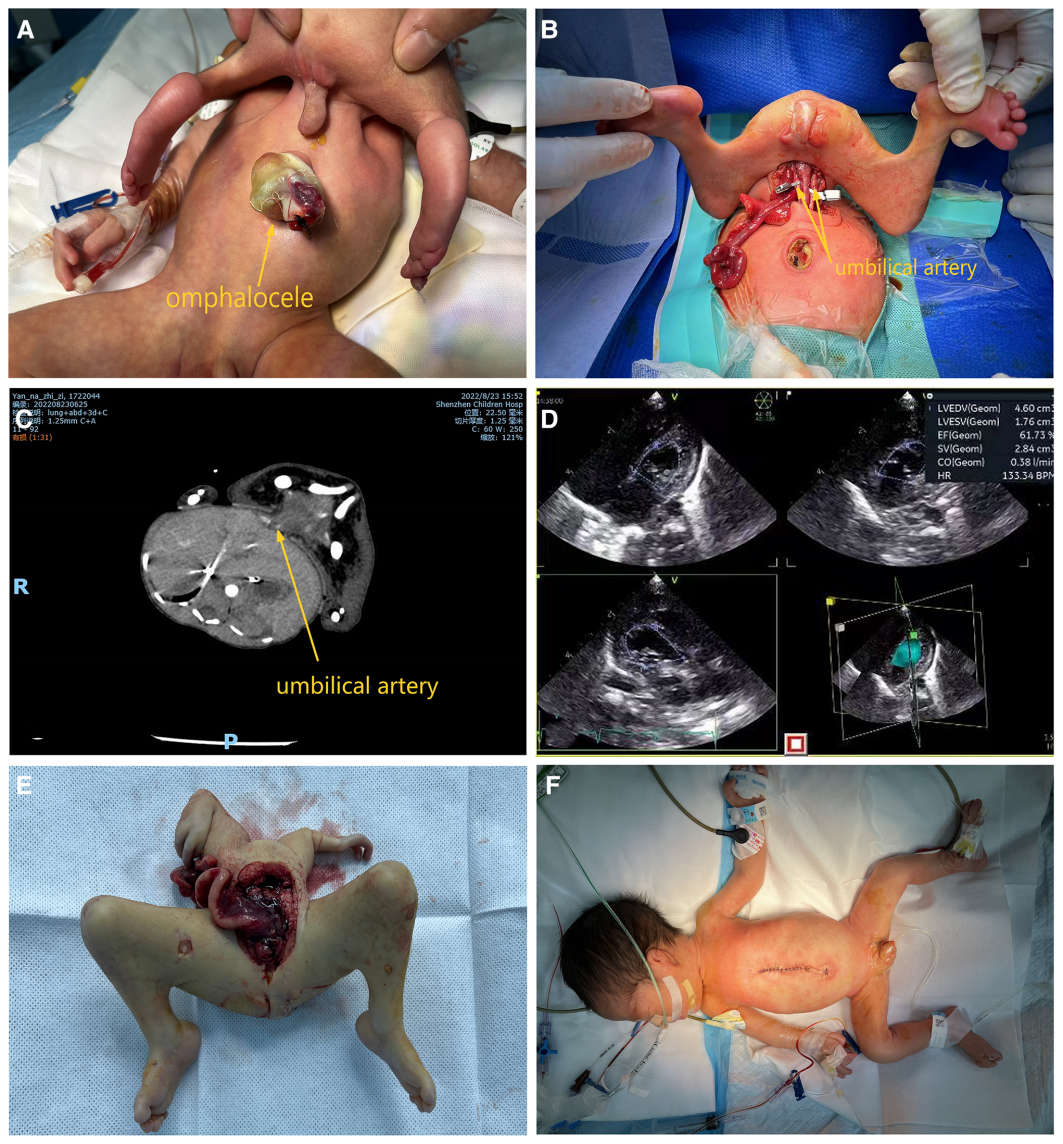
(**A**–**F**) Basic clinical datd of the children.

A channel approximately 1 cm in size was present above the umbilicus of the infant, with normal skin tissue outside the channel. The outer end of the channel was connected to a malformed fetus parasite approximately 40 × 10 cm in size after complete abduction. Male genitalia (urine was intermittently excreted) and an atrophied scrotum and buttocks were observed in the lower segment, and no anus was present. The parasite did not respond to stimulation and had no voluntary movement. The umbilical stump of the autosite protruded approximately outward and was covered with a cystic membrane; a gastrointestinal tract (GI) was visible, containing dark green contents (Figure 1A).

Ultrasound examination and echocardiography of the fetus on admission indicated (1) a moderately echogenic abdominal mass consistent with a parasitic fetus and (2) congenital heart disease with ventricular septal defect (myocardial), bidirectional shunt at the ventricular level, patent ductus arteriosus, bidirectional shunt at the aortic level, patent foramen ovale, left-to-right shunt at the atrial level, and pulmonary hypertension (Figure 1D).

A computed tomography (CT) scan indicated a parasitic fetus that was connected to the anterior abdominal wall of the autosite through the soft tissue in the pelvis by an omphalocele ending at the anterior margin of the left lobe of the liver. Congenital heart disease and locally poor demarcation between the left anterior margin of the liver and the parasitic fetus were also detected (Figure 1C).

**Figure 2:**
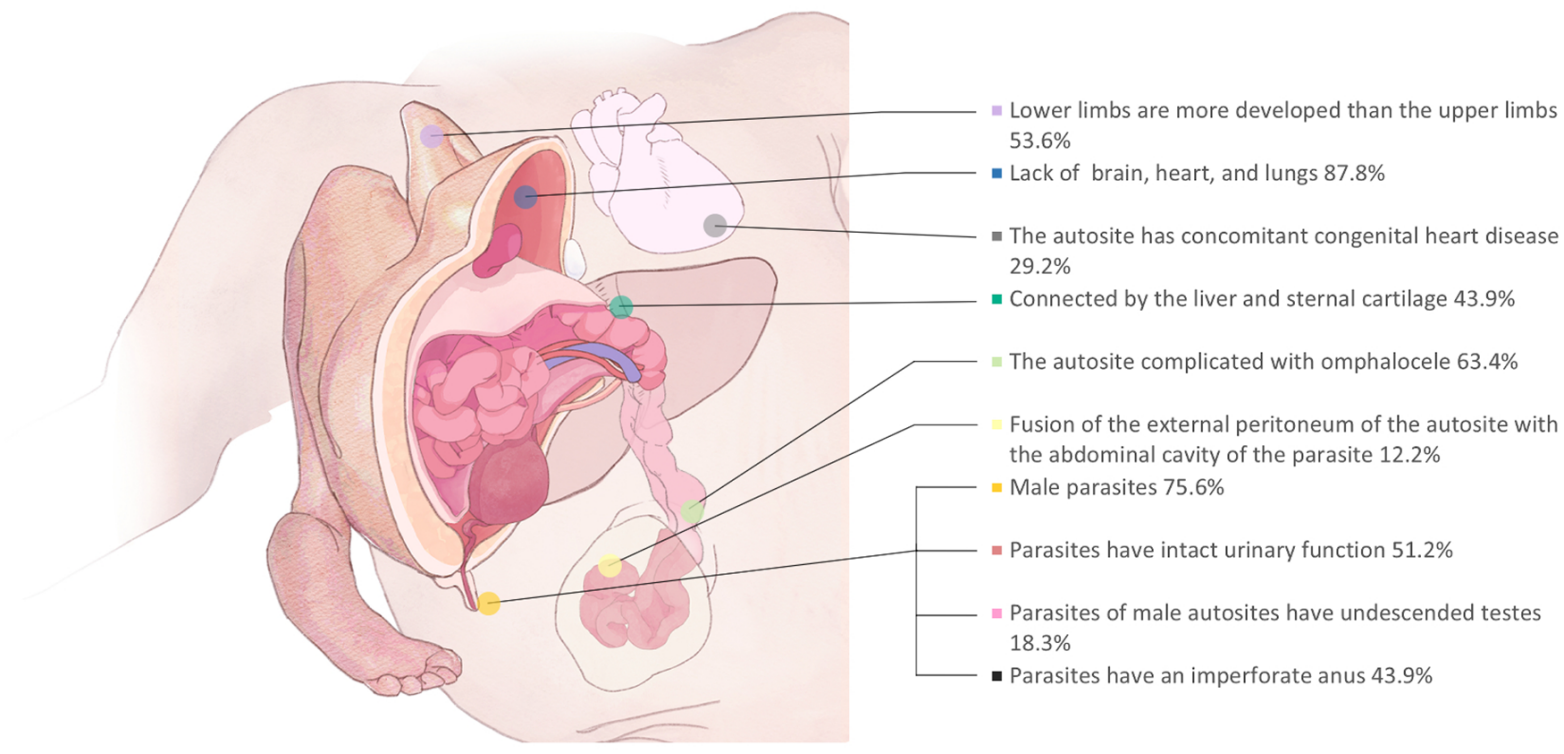
Disease characteristics of EHCTs reported in the literature: according to previous literature. Diseases EHCTs are characterized by the incidence of malformations, among which a more important part can be seen that a part of parasitic fetal intestine travels through the abdominal wall of the main body, which needs to be avoided during surgery.

Pre-operative treatment included cardiac and oxygen monitoring, warming, fluid replacement, and vitamin K1. After multidisciplinary consultation and preoperative preparation, parasite removal, omphalocele repair, umbilicoplasty, and abdominoplasty were performed under general anesthesia. The omphalocele membrane was incised, and the blind end of the GI tract was found to be connected to the membrane in the omphalocele. When separating adhesions, the ileocecal end was found to be connected to the blind end of the colon toward the proximal free parasitic GI tract, which was found to travel toward the surface of the autosite's liver, into the common channel, and then back into the parasite. A lumen was formed by the peritoneum between the surface of the liver of the autosite, the umbilicus of the autosite, and the common channel, which was not connected to the abdominal cavity of the autosite; the parasitic GI tract was located in this lumen (Figure 2). The skin was incised along the root of the connection between the parasite and autosite, and the subcutaneous and muscular layers were separated. The umbilical arteries on both sides of the parasite were found to be pulsating, and the urachus was connected to the parasite bladder. The urachus of the parasite was ligated and disconnected. On the dorsal side of the parasite, there was cartilage connected to the sternal cartilage of the autosite, and an artery and vein were seen running below the cartilage. The three arteries were occluded using vascular clips, and the parasitic fetus was found to be ischemic. After the veins were occluded using vascular clips, the arterial pressure, venous pressure, and blood gas analysis of the infant were stable, and vital signs were unchanged (Figure 1B). The artery and vein were individually ligated, and no change in arterial or venous pressure was observed. The sternal cartilage was severed, and the parasite was removed (Figure 1E). The stump of the urachus was traced into the abdominal cavity of the child and was found to extend toward the omphalocele, and the entire urachus was freed after resection. The abdominal wall and umbilical incisions were sutured separately to reconstruct the appearance of the umbilicus (Figure 1F). Pathological examination suggested a complete lack of muscular tissue and nerves in the limbs of the parasite.

After discharge, the infant was followed for more than 3 months, during which time growth and development were normal, feeding was well tolerated, and healing and morphology of the abdominal wall defect were satisfactory.

## Discussion

The first case of EHCT was reported in 1940 by Sarrelangue (2), and a database search of English and non-English language literature revealed fewer than 50 cases to date (Supplementary Table S1). EHCTs have a striking appearance, often combined with massive limbs. However, the connecting structures are relatively rudimentary, and most are connected only by blood vessels and cartilage (Figure 2). There was only one case where the parasite was connected to the autosite intestine (3). Therefore, most cases may potentially avoid organ dissection, intestinal resection, and anastomosis, which also reduces the risk of postoperative intestinal adhesion, intestinal obstruction, and organ failure. Therefore, surgical intervention of EHCT is not complicated. And we found a high postoperative survival rate (38/41, 92.6%, Supplementary Table S1). However, the etiology of EHCTs is still not clear. Prenatal evaluation of these anomalies is necessary, and a better understanding of EHCTs may prevent unnecessary termination of pregnancy. With parental consent, amniocentesis is recommended for further monitoring of fetal chromosome karyotype, other associated congenital diseases should be considered.

Because the parasite is often large and irregular in size, a cesarean delivery is recommended to avoid difficult delivery and birth injury. The initial diagnosis by prenatal ultrasound and the reliance on postnatal ultrasound, CT, and magnetic resonance imaging can provide a clear picture of the blood supply and the type of connection (4), which helps improve the success rate of surgery and reduce postoperative complications.

EHCTs present a high incidence of congenital heart disease, which has also been hypothesized by Ugarte et al. as arising from the hemodynamics necessary for the autosite to support the parasite (5, 6). Therefore, immediate postpartum surgery is not necessary. It is necessary to perform a detailed imaging examination and monitor cardiac function before surgery. Children with omphalocele (63.4%) should be closely arranged for preoperative examination after delivery, and attention should be paid to the protection of the capsule. Care must be taken during the perioperative period to avoid compression of autosite respiratory functions by the parasite and to monitor any changes in oxygen saturation (7). In addition, if the main body flap is insufficient during EHCT surgery, the surgical incision should be as close to the parasite as possible, so that a sufficient flap can be preserved.

As the parasite blocks and obscures its site of attachment to the autosite, the parasite must be moved intraoperatively to expose the surgical site, and the anesthesiologist must be aware of the need for improved tracheal intubation and blood pressure management. The vascular supply of the parasite has been reported to originate from the liver, the left internal mammary artery, vessels in the abdominal wall, and the ductus venosus (1). The arteries should be gradually ligated during surgery to slowly increase the amount of blood returned to the heart, thereby preventing heart failure and pulmonary edema (8). The artery and vein can also be gradually clamped first, paying close attention to central venous pressure, changes in arterial and venous pressure, and other vital signs of the autosite, and then ligated after stabilization.

In EHCTs with omphalocele, care must be taken to elucidate the site of the fusion of the autosite with the abdominal wall of the parasite and the contents of the omphalocele, such as the GI tract. We identified five reported cases of herniation of the parasitic intestinal canal into the omphalocele of the autosite. There have been reports of reoperation for abdominal wall infection secondary to residual parasitic GI tract located in the abdominal wall of the patient from the initial operation (9). Therefore, if intraoperative exploration reveals the presence of the GI tract at the connection site, careful dissection is required to avoid overlooking the parasite GI tract or damaging the autosite GI tract.

Hopkins et al. (10) reported a case of a retroperitoneal parasitic fetus with postoperative recurrence of an endodermal sinus tumor and suggested that the parasitic fetus must be completely excised, and postoperative monitoring should be emphasized in patients with the anomaly. Therefore, routine postoperative abdominal imaging reexamination and monitoring of serum tumor markers are also considered necessary. However, unlike a retroperitoneal parasitic fetus, EHCTs have a skin-covered parasite with more extensive mature organ differentiation. No cases of malignant transformation have been reported.

## Conclusions

EHCTs usually present rudimentary structures at the connection site between the autosite and the parasite that are not closely associated with organ function, thus enabling successful treatment by surgery. Intrauterine evaluation of such malformations, careful prenatal assessment of the organs, and content assessment of the autosite-parasite connection site using ultrasound are required to develop a postpartum treatment strategy to prevent unnecessary termination.

## Data Availability

The original contributions presented in the study are included in the article/**Supplementary Material**, further inquiries can be directed to the corresponding authors.
