# Supplementary material for: Case report: Epigastric heteropagus twins and literature review
Source: Front Pediatr. 2023 Apr 12;11:1088480. doi: 10.3389/fped.2023.1088480 (PMC10132136; doi:10.3389/fped.2023.1088480)
Supplement: Supplementary file 1 [file Table1.pdf]

Supplementary Material

Supplementary Table 1

No.	Publication date	Country	Author	Tissues in conjoined region	Organs in parasite	Sex	Undescended testicle in parasite	Parasite able to pass urine	Imperforate anus in parasite	Lower limbs more developed than upper limbs	Voluntary movement	Sensation	Conjoined GI tracts	Omphalocele	Congenital defects in autosite	Outcome
1	1946	Mexico	Sarrelangue et al.	Liver	Limbs, GI tract, bladder, atrophied liver	M	ID/NS	+	+	+	-	-	-	-	CHD	
2	1969	Poland	Poradowska et al.	ID/NS	Limbs	M	-	ID/NS	ID/NS	+	ID/NS	ID/NS	-	+	CHD (CoA, single atrium)	Autosite died of circulatory disorder
3	1978	Nigeria	Mabogunje et al.	Sternum	Limbs, kidney, ureter, bladder, GI tract	F	ID/NS	+	ID/NS	+	-	ID/NS	-	-	ID/NS	CNC
4	1982	South Africa	Cywes et al.	Sternum	Limbs	M	Unilateral undescended testis	+	+	+	-	-	+	+	Esophageal atresia, CHD	CNC

															(VSD), Meckel’s diverticulum	
5	1986	Italy	Nasta et al.	Small bowel	Limbs	M	Unilateral undescended testis	+	+	+	-	-	-	+	CHD (VSD), esophageal atresia	CNC
6	1986	India	Surendran et al.	ID/NS	Limbs, rectum, bladder	M	+	-	+	+	ID/NS	ID/NS	-	+	Club foot	CNC
7	1986	India	Pattabiraman et al.	ID/NS	Limbs, liver, small bowel, kidney, ureter, bladder, both testes	M	ID/NS	+	ID/NS	+	ID/NS	ID/NS	-	+		CNC
8	1992	India	Biswas et al.	Liver	Bilateral upper limbs and unilateral lower limbs, face, GI tract	M	ID/NS	ID/NS	ID/NS	ID/NS	ID/NS	ID/NS	-	+		CNC
9	1993	India	Chadha et al.	Abdominal wall vessels	Limbs, kidney, bladder, atrophied GI tract	M	-	+	+	+	-	-	-	+	CHD	CNC

10	1994	Turkey	Sarihan et al.	ID/NS	Limbs, liver has two reduced kidneys and ureters, an empty bladder	M	+	+	+	+	-	ID/NS	-	+	CHD (VSD), inguinal hernia	CNC
11	1995	Bangladesh	Saha et al.	ID/NS	Limbs, pelvis, sternum, kidney, ureter, seminal vesicle, prostate	M	-	+	+	+	-	ID/NS	-	-		CNC
12	1996	South Korea	Hwang et al.	ID/NS	Bilateral lower limbs, kidney, bladder	M	+	ID/NS	+	+	-	-	-	+	CHD (single ventricle，PDA）	CNC
13	1998	India	Jain et al.	GI tract	Limbs, two vertebrae, kidney, ureter, reduced bladder	M	+	+	+	+	-	-	-	+		CNC
14	1998	Russia	Bairov et al.	ID/NS	ID/NS	M	ID/NS	ID/NS	ID/NS	ID/NS	ID/NS	ID/NS	ID/NS	-		CNC

15	1999	India	Borah	ID/NS	ID/NS	M	ID/NS	ID/NS	ID/NS	ID/NS	ID/NS	ID/NS	ID/NS	+		CNC
16	1999	Turkey	Kamak et al.	Pelvis	Limbs, bladder, kidney, ureter	M	+	+	+	+	-	-	-	+		Complete resection, postoperative wound infection, complete recovery after infection controlled
17	1999	China	Zhou et al.	Liver	Incompletely developed skull, univentricular heart, single adrenal, single lung, ureter, gonads, GI tract	M	+	-	ID/NS	ID/NS	ID/NS	ID/NS	-	+	CHD (ASD)	CNC
18	2000	Turkey	Özcan et al.	Sternum, liver	Limbs, heart, lungs, pancreas, gallbladder	M	Unilateral undescended testis	-	+	+	+	-		-		Died postoperatively
19	2000	China	Mao	Liver	Bilateral lower limbs	F	ID/NS	+	ID/NS	ID/NS	ID/NS	ID/NS	-	+		CNC

20	2001	France	Petit et al.	ID/NS	Bilateral lower limbs, GI tract	M		+	+	+	-	ID/NS	-	+		CNC
21	2001	Brazil	Cury et al.	Sternum	Liver	F	ID/NS	+	ID/NS	+	-	ID/NS	-	-		CNC
22	2001	India	Gupta et al.	Sternum	Kidney, ureter, bladder, GI tract	M	ID/NS	ID/NS	ID/NS	ID/NS	ID/NS	ID/NS	ID/NS	+	Sternal defects	CNC
23	2002	China	Zhang et al.	Sternum	Testis, cartilage, glands, hair, glomerulus-like tissue	M	+	-	ID/NS	ID/NS	ID/NS	ID/NS	-	+	Polydactyly	CNC
24	2003	China	Yang et al.	Liver	Right kidney, bladder, urethra, GI tract	M	-	+	ID/NS	+	ID/NS	ID/NS	-	+		CNC
25	2004	India	George et al.	Sternum, GI tract, liver, gallbladder	Bilateral lower limbs, external genitalia, liver	M	+	-	ID/NS	ID/NS	-	-	-	-	Club foot	Incomplete resection, infection of residual autosite abdominal wall and GI tract, complete

																recovery after re-operation
26	2005	India	Bhansali et al.	Gastrointestinal tract, sternum	Bilateral lower limbs, GI tract	F	ID/NS	ID/NS	+	ID/NS	-	ID/NS	-	+		Complete resection, died of sepsis on postoperative day 3
27				ID/NS	Two limb buds	M	ID/NS	ID/NS	ID/NS	ID/NS	-	ID/NS	ID/NS	+		CNC
28				ID/NS	Bilateral lower limbs	F	ID/NS	ID/NS	ID/NS	ID/NS	-	ID/NS	ID/NS	-		CNC
29	2005	Brazil	Ribeiro et al.	ID/NS	Skull, heart, bilateral kidneys, GI tract	M	-	-	-	-	-	ID/NS	-	-	Unilateral renal agenesis	CNC
30	2006	USA	Hager et al.	Upper thorax	GI tract, liver	F	ID/NS	+	+	+	-	-		-	Spinal injury, CHD	CNC
31	2006	Japan	Shibata et al.	ID/NS	Skull	ID/NS	ID/NS	ID/NS	ID/NS	ID/NS	ID/NS	ID/NS	ID/NS	-		Electively terminated
32	2007	Austria	Hager et al.	ID/NS	Skull, joints, synovial membrane, one bone and incomplete developme	F	ID/NS	ID/NS	ID/NS	ID/NS	ID/NS	ID/NS	ID/NS	-	CHD(VSD)	CNC

					nt of esophagus, stomach, jejunum, trachea, and lung											
33	2008	USA	Satter et al.	Gastrointestinal tract, liver	GI tract, liver	F	ID/NS	+	-	ID/NS	-	-	-	-		CNC
34	2008	China	Dai et al.	Sternum, liver	Kidney, ureter, bladder	M	-	+	-	ID/NS	-	-	-	+	CHD (ASD)	Complete resection, mild ventral hernia
35	2009	China	Hu et al.	ID/NS	GI tract, kidney, bladder, ureter	M	+	+	+	+	-	-	-	-	CHD (ASD)	CNC
36	2010	China	Xing et al.	Sternum	Bilateral lower limbs, hip bone, GI tract, kidney, bladder	M	+	+	+	+	-	-	-	+		CNC
37	2010	Nigeria	Abubakar et al.	Sternum	Bladder, gonads	M	+	+	+	+	-	-	-	+		CNC
38	2011	China	Gu et al.	ID/NS	Bilateral lower limbs	M	ID/NS	ID/NS	ID/NS	ID/NS	-	-	-	+		CNC

39	2011	China	Xu et al.	ID/NS	Bilateral lower limbs	M	ID/NS	ID/NS	ID/NS	ID/NS	ID/NS	ID/NS	-	+		CNC
40	2018	China	Song et al.	Liver	Liver	ID/NS	ID/NS	ID/NS	ID/NS	ID/NS	ID/NS	ID/NS	ID/NS	+		
41	2020	China	Xu et al.	ID/NS	ID/NS	M	+	+	+	+	-	-	-	-	ID/NS	CNC

ID/NS: insufficient data, not studied, or not applicable

CHD, congenital heart disease (not elaborated in article); ASD, atrial septal defect; VSD, ventricular septal defect; PDA, patent ductus arteriosus; PA, pulmonary atresia; CoA, coarctation of aorta; GI, gastrointestinal

CNC: Complete resection, no complications, complete recovery
